# Enhanced Stability of Multi-Functionalized Gold Nanoparticles and Potential Anticancer Efficacy on Human Cervical Cancer Cells

**DOI:** 10.3390/biomedicines13081861

**Published:** 2025-07-31

**Authors:** Aurora Mocanu, Madalina Anca Ujica, Ossi Horovitz, Gheorghe Tomoaia, Olga Soritau, Cristina Teodora Dobrota, Cristina Roxana Popa, Attila Kun, Horea-Rares-Ciprian Benea, Ionel Marius Mang, Gheorghe Borodi, Viorica Raischi, Marius Roman, Lucian Cristian Pop, Maria Tomoaia-Cotisel

**Affiliations:** 1 Research Center of Excellence in Physical Chemistry, Faculty of Chemistry and Chemical Engineering, Babeş-Bolyai University, 11 Arany Janos St., RO-400028 Cluj-Napoca, Romania; mocanu.aurora@gmail.com (A.M.); madalina.ujica@ubbcluj.ro (M.A.U.); ossihor@yahoo.com (O.H.); cristina.dobrota@ubbcluj.ro (C.T.D.); popa.roxana90@yahoo.com (C.R.P.); attila.kun@ubbcluj.ro (A.K.); ionelmang@yahoo.com (I.M.M.); or vioricalana@gmail.com (V.R.); 2 Department of Orthopedics and Traumatology, Iuliu Hatieganu University of Medicine and Pharmacy, 47 General Traian Mosoiu St., RO-400132 Cluj-Napoca, Romania; tomoaia2000@yahoo.com (G.T.); benea.horea@umfcluj.ro (H.-R.-C.B.); 3 Academy of Romanian Scientists, 3 Ilfov St., RO-050044 Bucharest, Romania; 4 Ion Chiricuta Oncologic Institute of Cluj-Napoca, 34-36 Republicii St., RO-400015 Cluj-Napoca, Romania; olgasoritau@yahoo.com; 5 National Institute for Research and Development of Isotopic and Molecular Technologies, 65-103 Donath St., RO-400293 Cluj-Napoca, Romania; borodi@itim-cj.ro; 6 Institute of Physiology and Sanocreatology, State University of Moldova, Academy 1 St., 2028 Chișinău, Moldova; 7 INCDO-INOE 2000, Research Institute for Analytical Instrumentation, 67 Donath St., RO-400293 Cluj-Napoca, Romania; marius.roman@icia.ro

**Keywords:** multi-functionalized gold nanoparticles, green synthesis of gold nanoparticles, drug delivery nanoparticles, doxorubicin, resveratrol, piperine, icariin, cervical cancer

## Abstract

**Objectives:** In this research study, we introduce a novel approach to develop an innovative nanocarrier system comprising gold nanoparticles (GNPs) loaded with doxorubicin (D) in combination with natural molecules, such as trans-resveratrol (R), piperine (P), and icariin (Ic), against human cervical cancer. The final objective is to improve the anticancer efficacy of doxorubicin on HeLa and CaSki cell lines. **Methods:** Resveratrol was also used for the synthesis of GNP_R1 nanoparticles. Multi-functional GNPs loaded with D, R, P, and Ic (e.g., GNP_R1@D/R/P/Ic) were successfully prepared and fully characterized by SPR, TEM, HR-TEM, XRD, AFM, DLS, and zeta potential. They were investigated for in vitro stability in various biological media. The cytotoxicity activity was tested on HeLa and CaSki cell lines, using the MTT assay, for their applications as anticancer agents. **Results:** Our results demonstrate that the novel multi-functional GNPs (such as GNP_R1@D/R and GNP_R1@D/R/P/Ic) can effectively target the cervical cancer cells, improving the bioavailability of therapeutic agents and enhancing their cytotoxicity against cervical cancer cells. In vitro assessments demonstrated that the multi-functional GNPs exhibited improved stability and potential anticancer efficacy on human cervical cancer cells. **Conclusions**: The described strategy connects the benefits of biomolecules with functional nanoparticles toward the development of various GNP_R1@D/R/P/Ic nanocarriers for their applications as anticancer agents against human cervical cancer. This study provides compelling evidence that the innovative nanoparticles can enhance the therapeutic efficacy of doxorubicin against cervical cancer and offer a more advantageous alternative compared to doxorubicin monotherapy.

## 1. Introduction

Cervical cancer, triggered mainly by persistent infection with human papillomavirus (HPV) associated with chronic inflammatory processes, is one of the most prevalent cancers in women. The standard treatment for cervical cancer involves surgery, chemotherapy, and/or radiotherapy, with their related adverse effects and high chances of recurrence. Therefore, there is a need to develop new treatment strategies to improve the effectiveness of chemotherapy. Recently, nanotechnology has attracted increasing researchers’ attention using nanoparticles in the treatment of human cervical cancer. This research is an active field for drug delivery, with a focus on developing excellent nanocarriers with advanced physical and chemical properties [1].

At this moment, the focus lies mainly on gold nanoparticles (GNPs), which are the most investigated employing several surface engineering strategies [2,3,4,5,6,7,8]. The GNPs’ combination with other active anticancer molecules results in multi-functional nanoparticles [2]. These advanced nanoparticles possess both the chemical properties of GNPs and the functions of the coupling compounds. Doxorubicin, D, is considered an effective chemotherapeutic drug; however, it is also frequently linked to drawbacks, such as increased toxicity to healthy tissue or the potential to induce multidrug resistance [7]. Therefore, the goal of this research is to enhance the effectiveness of this drug at concentrations smaller than those used clinically. This can be achieved by using natural molecules, such as trans-resveratrol (R), piperine (P), and/or icariin (Ic), with demonstrated anticancer effects [9,10,11,12] and gold nanoparticles [7,13,14,15,16,17,18,19,20]. This innovative approach can reduce doxorubicin’ s cardiotoxic adverse effects [13,14] while increasing its accumulation in tumors, thereby initiating new cancer therapies.

It was demonstrated that the action of doxorubicin can be potentiated by its conjugation (complexation) with gold nanoparticles, GNPs, constituting a novel strategy to facilitate its penetration into the cancer cells [15,16,17,18,19,20,21,22,23,24,25,26]. When its complexation is carried out with gold nanoparticles, GNPs [27,28,29,30,31], the release of doxorubicin is potentiated by the acidic environment, which can be also found in physiological conditions [32,33,34]. The adsorption of doxorubicin on GNPs is realized, most likely, by its protonated amino group in a slightly acidic environment (pH 6) [35,36]. These findings allow for the development of new techniques that use nanoparticles to improve the permeability and retention of anticancer medicines in cancer cells. These techniques offer high potential for combining numerous medications. The negative effects of D can be reduced with regulated medication distribution via nanocarriers. Recently, resveratrol (R), piperine (P), and/or icariin (Ic) have been widely used as active agents against cancer cells.

Resveratrol is a polyphenol produced by plants [37] that exhibits cardioprotective effects through various mechanisms. These mechanisms include antioxidant activity, free radical scavenging, and the inhibition of lipid peroxidation. R can form a self-assembled coating layer on GNPs, which has demonstrated biocompatibility, making them a suitable candidate for enhancing drug effectiveness [7,38,39]. Numerous studies have confirmed the many beneficial effects of resveratrol on the living organism, for instance, the anti-inflammatory action, modulation of lipid metabolism, and inhibition of platelet aggregation [40,41]. Even on its own, resveratrol has a slight anticancer effect by sensitizing cancer cells [42,43]. It has been shown to reduce cellular oxidative stress and inflammation [42,44] and to modulate the activity of doxorubicin on cervical cancer cells, HeLa and CaSki, causing cell death by apoptosis [7,45]. Resveratrol also has the ability to reduce the side effects of doxorubicin, leading to protection against both the initiation and the development of tumors [43]. Sun et al. (2021) reported that the antitumor effect of resveratrol was exhibited due to the inhibition of the action of HPV-specific genes (*E6* and *E7*) by inducing apoptosis and reducing both the viability and mitotic index of cervical cancer cell lines [46].

Piperine, P, is an alkaloid extracted from *Piper longum* and *Piper nigrum* that has been used for a long time [47,48] for its antioxidant, anti-inflammatory, and immunomodulatory effects [49]. It has both anticancer and protective roles on healthy cells, by rejuvenating senescent cells [50]. Piperine can modulate the bioactivity of resveratrol [51,52], and in combination with resveratrol, either as a physical mixture, R:P, or as an RP co-crystal, it increases the sensitivity of cancer cells, promoting cell death by apoptosis [53].

Icariin, Ic, is the main active ingredient in a Chinese anti-inflammatory plant, *Epimedium.* Icariin was reported to have anti-inflammatory, anti-oxidative, anti-depression, and anticancer effects. Icariin has clinical applications in neurodegenerative and cardiovascular diseases, osteoporosis, and cancer [54]. Icariin is presented as an active participant in the increased intracellular accumulation of doxorubicin [55,56,57]. Because these chemicals are poorly soluble in water, they must be made more bioavailable. One possibility is to employ them in various nano-formulations, which would allow for a reduction in active dosages of doxorubicin while simultaneously providing a longer release time to target tissues.

According to the described features of icariin, piperine, resveratrol, and doxorubicin, these compounds are expected to form novel nanocarriers with GNPs, as noted GNP_R1@D/R/P/Ic. This concept is based on a novel combinatorial therapeutic approach for cervical cancer treatment. We use two models of cervical cancer in this study (HeLa and CaSki cells). Cell viability was measured in response to a variety of nanocarriers based on GNPs functionalized with resveratrol, piperine, and icariin, with or without doxorubicin. Another comparative study was performed on HeLa and CaSki cells’ viability after 24 and 48 h, between free biocompounds and GNPs functionalized with these biocompounds, each loaded with the same dose of doxorubicin of 2 µg/mL.

All these biocompounds, R, P, and Ic, have antitumor effects. Their biological effects on tumor cells are mainly to induce apoptosis through different signaling pathways. Their low water solubility brings the need to increase their bioavailability. One of the solutions is their use in different nano-formulations, which would facilitate a decrease in active doses but also a prolonged release at the target tissues.

The major precursor used in the manufacture of GNPs is chloroauric acid, which is reduced with a variety of agents. In chemical synthesis, Au^3+^ is reduced to Au^0^ using sodium borohydride or trisodium citrate. Biological synthesis employs diverse plant or bacterial extracts [58,59,60,61]. Resveratrol is a reducing agent commonly utilized in green synthesis [7,38,59,60,62,63]. Following synthesis, the dispersion of gold nanoparticles coated with a resveratrol layer, denoted as GNP_R, is purified by centrifugation and washed under circumstances similar to those described in the literature [64,65,66]. The acquired GNP pellet is redispersed in bi-distilled water, saline solutions, PBS (pH ~ 7.4), or cell culture media (pH 7.2–7.4), resulting in purified gold nanoparticles (noted GNP_R1) in colloidal solutions.

Previously [7], we developed anticancer carriers as GNP_R@D/R complexes, containing doxorubicin (D) and trans-resveratrol (R). They were used on HeLa and CaSki cells. The results showed that GNPs and resveratrol improved doxorubicin’s antitumor efficacy, mainly through the apoptotic mechanism.

In this study, we report on effective anticancer nanoscale carriers based on gold nanoparticles, such as GNP_R1, functionalized with a standard drug, doxorubicin (D), and natural molecules resveratrol (R), piperine (P), and/or icariin (Ic), each with demonstrated anticancer activity. The aim of this study was mainly to explore the stability of multi-functional gold nanoparticles, such as advanced GNP_R1@D/R/P/Ic nanocarriers, and their anticancer efficacy on human cervical cancer—especially the HeLa and CaSki—cell lines. The key approach is associated with dose–response studies on chosen cell lines and predicts therapeutic doses established by in vitro cytotoxicity versus untreated cells. 

## 2. Materials and Methods

### 2.1. Materials

Tetrachloroauric acid trihydrate (HAuCl_4_·3H_2_O) 99.5% (Merck, Darmstadt, Germany), trans-resveratrol ≥ 99% (HPLC assay, from Sigma-Aldrich, Buchs, Switzerland), and NaOH reagent grade ≥ 98% (Merck KGaA, Darmstadt, Germany) in aqueous solutions prepared with bi-distilled deionized water were used in the preparation of gold nanoparticles (GNPs). The following were used in the functionalization of GNPs: doxorubicin hydrochloride (about 98%) from Sigma-Aldrich Chemie GmbH (Munich, Germany), piperine ≥ 98% (HPLC assay) from AlfaAesar (Karlsruhe, Germany), and icariin, analytical standard (≥94%), from Sigma-Aldrich (Steinheim, Germany). Dimethyl sulfoxide (DMSO) was purchased from Sigma-Aldrich (Schnelldorf, Germany). Dulbecco’s phosphate-buffered saline (PBS), without CaCl_2_ and MgCl_2_, (pH 7.4), was purchased from AlfaAesar (Karlsruhe, Germany). MTT tetrazolium salt (3-(4,5-dimethylthiazol-2-yl)-2,5-diphenyltetrazolium bromide) ≥ 98% was acquired from Sigma-Aldrich (Buchs, Switzerland).

### 2.2. Synthesis of Gold Nanoparticles, GNPs

GNPs were synthesized by the green reduction of HAuCl_4_ with resveratrol [7,38]. In brief, a freshly prepared solution of 50 mg resveratrol in 20 mL 0.02 M NaOH was added to 200 mL 10^−3^ M HAuCl_4_ in bi-distilled water, under steady stirring at 500 RPM for 10 min at room temperature. The gold nanoparticles, formed by reducing HAuCl_4_ with resveratrol and noted GNP_R, were obtained in the initial gold concentration of 0.91 mM (179 mg/L Au) in colloidal solution. They were centrifuged in the same manner as shown in the literature [64,65,66] to eliminate the secondary reaction products, obtained during their synthesis. Therefore, the gold nanoparticles obtained by synthesis were purified by centrifugation (at around 10,000 RPM) using a Hettich Universal 320R centrifuge, Beverly, MA, USA, in optimal experimental conditions for about 20 min at 5 °C, with a relative centrifugation force, RCF, of 8000× *g*.

These centrifuged GNP_R particles were washed with bi-distilled water of pH 5.6. Then, they were centrifuged again and redispersed in bi-distilled water to produce a final colloidal solution (referred to as GNP_R1) with the same gold concentration as in the initial solution of synthesized GNP_R, as confirmed by UV-VIS spectroscopy. This GNP_R1 colloidal solution was kept at 4 °C, in the dark, to prolong its stability until use. This GNP_R1 colloidal solution through dilution with PBS (pH 7.4) or with cell culture media, such as DMEM (pH 7.2–7.4), was used in cancer cell culture media.

### 2.3. Functionalization of Gold Nanoparticles

The GNP_R1 colloidal solution was further used in functionalization of GNP_R1 particles with selected natural compounds, resveratrol, piperine, and icariin, which are recognized as molecules that target cervical cancer cells. Thus, a rational combination strategy was developed for the enhancement of multi-functional gold nanoparticles’ stability and for synergistic multi-target improvement in doxorubicin anticancer efficacy on human cervical cancer cells.

Functionalization of resveratrol-covered gold nanoparticles, GNP_R1, with more resveratrol leads toward the development of GNP_R1@R, which is primarily ruled by physical adsorption of R molecules within the R layer on gold nanoparticles. The high stability of this self-assembled R layer on Au surface is described through intermolecular hydrogen bonds and electrostatic interactions, without forming chemical bonds with the GNPs [7]. This strategy to increase the number of biocompounds within the self-assembled R layer on Au surface proved successful. Further, GNP_R1 was functionalized with R, P, D, and Ic, resulting in GNP_R1@R/P/D/Ic nanocarriers that showed superior anticancer activity, attributed to an optimal cellular uptake after 24 h incubation, or even more at 48 h incubation, as demonstrated in Section 3.

**Figure 1:**
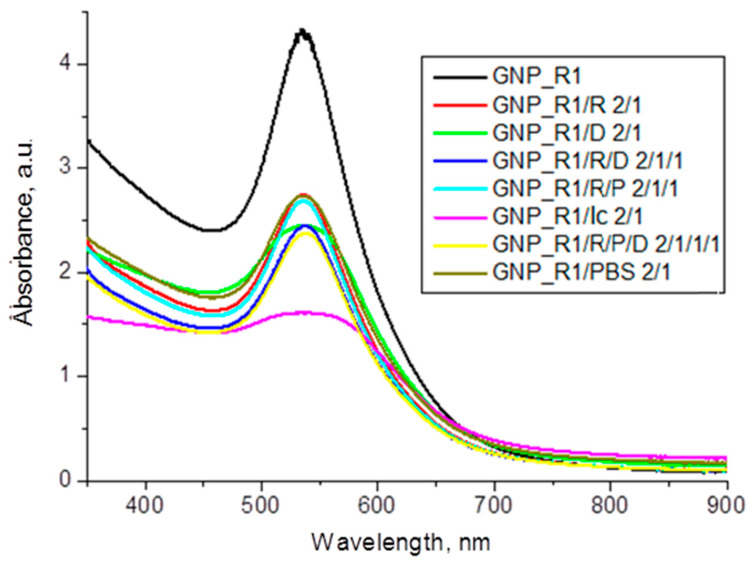
UV-VIS spectra of GNP_R1 (Au 179 mg/L) aqueous dispersion, and mixtures GNP_R1/R 2/1 *v*/*v* ratio (119.3 mg/L Au, 10 mg/L R); GNP_R1/D 2/1 *v*/*v* ratio (119.3 mg/L Au, 14 mg/L D); GNP_R1/R/D 2/1/1 *v*/*v*/*v* ratio (89.5 mg/L Au, 7.5 mg/L R, 10.5 mg/L D); GNP_R1/R/P 2/1/1 *v*/*v*/*v* ratio (89.5 mg/L Au, 7.5 mg/L R, 10 mg/L P); GNP_R1/Ic 2/1 *v*/*v* ratio (119.3 mg/L Au, 33.3 mg/L Ic); GNP_R1/R/P/D 2/1/1/1 *v*/*v/v*/*v* ratio (71.6 mg/L Au, 6 mg/L R, 8 mg/L P, 8.4 mg D); and GNP_R1/PBS 2/1 *v*/*v* ratio (119.3 mg/L Au).

To address these challenges, various solutions with different proportions of anticancer compounds, namely, doxorubicin hydrochloride, resveratrol, piperine, and/or icariin, as well as mixtures of these solutions, were added to GNP_R1 aqueous colloidal solution (0.91 mM Au) at 25 °C (or at room temperature), while thoroughly stirring, in the dark, for around 30 min. All solutions used in this study are given in Table 1. Icariin, which is not soluble in water, was solubilized in dimethyl sulfoxide (DMSO, 11 mg/10 mL), and then 100 mL PBS was added to the solution. Several examples are given in the caption of Figure 1, as well as those further diluted with PBS solution to obtain the concentrations given in Table 2 and Table 3, for the cellular investigations. The mixtures were then maintained at the chosen temperature while thoroughly stirring, in the dark, until used in physicochemical investigations or in cellular treatment (up to 1 h). Identification of surface functional group coating GNPs was performed by Fourier transform infrared spectroscopy (FTIR) as shown earlier [7], and the results are not given here.

### 2.4. Cell Lines

Certified cell lines of HeLa and CaSki cervical carcinoma cells were used in this study. Cell lines were provided by the European Collection of Authenticated Cell Cultures (ECACC) and purchased from Sigma-Aldrich.

Adult mesenchymal stem cells (MSCs) derived from dental follicles, noted dental follicle MSCs (DF MSCs), were investigated as healthy cells [67] in this study; isolation and working protocols were approved by the Ethical Committee of the Iuliu Hatieganu University of Medicine and Pharmacy, Cluj-Napoca, registration number 292/6.05.2011.

The cultivation medium was formulated as follows: HeLa cells were cultivated with Dulbecco’s modified Eagle (DMEM) medium, low glucose, supplemented with 10% fetal calf serum (FCS), 2 mM L-glutamine, 1% antibiotic, and 1% non-essential amino acids (NEAs) at 37 °C in 5% CO_2_ humidified air; for CaSki cells, the cultivation medium was RPMI-1640 medium supplemented with 10% fetal calf serum (FCS), 2 mM L-glutamine, and 1% antibiotic; all Sigma-Aldrich reagents.

Dental follicle MSCs were cultivated Dulbecco’s modified Eagle medium high glucose/F-12HAM containing 15% fetal calf serum (FCS, Sigma), 2 mM L-Glutamine, 1% antibiotics, 1% non-essential amino acids (NEAs), 55 μM beta-mercaptoethanol, and 1 mM natrium pyruvate [67]; all Sigma-Aldrich reagents. (For the effects of functionalized GNPs on healthy dental follicle mesenchymal stem cells (DF MSCs), see the Supplementary Material).

### 2.5. Characterization Methods

#### 2.5.1. The UV-VIS Absorption Spectra

The UV-VIS absorption spectra were measured with a Jasco UV/Vis V650 spectrophotometer, with a wavelength range from 190 to 900 nm; Jasco Corporation, Tokyo, Japan. 

#### 2.5.2. TEM Images 

TEM images were taken with a transmission electron microscope, JEOL—JEM 1010 (JEOL, Tokyo, Japan). The GNP colloidal solutions were deposited on carbon-coated copper grids.

#### 2.5.3. High-Resolution TEM (HR-TEM) Images 

High-resolution TEM (HR-TEM) images were obtained from an FEI Tecnai F20 field emission HR-TEM operating at an accelerating voltage of 200 kV; FEI, Hillsboro, OR, USA. Electron micrographs were recorded on an Eagle 4k CCD camera [68].

#### 2.5.4. Atomic Force Microscopy, AFM Images 

Atomic Force Microscopy, AFM images were obtained on GNP films adsorbed on glass from the colloidal solutions. Images were acquired by means of an AFM JEOL 4210, operated in tapping mode, with standard cantilevers with silicon nitride tips (frequency of resonance: 200–300 kHz; spring constant 17.5 N/m). The AFM images (2D and 3D topographies and phase images) were processed by the standard procedures [69,70,71].

#### 2.5.5. Zeta (ξ-) Potential and Dynamic Light Scattering (DLS) Measurements 

Zeta (ξ-) potential and dynamic light scattering (DLS) measurements were performed on the colloidal gold solutions using the Zetasizer Nano-ZS90, Malvern Panalytical, London, UK.

#### 2.5.6. X-Ray Diffraction (XRD) Studies 

X-Ray Diffraction (XRD) studies used a Bruker D8 Advance diffractometer (Bruker, Billerica, MA, USA), in Bragg–Brentano geometry, equipped with an X-ray tube with copper (Kα line, wavelength 1.541874 Å) [71].

#### 2.5.7. MTT Viability Assay

To perform the MTT test, the cells were trypsinized and counted with a hemocytometer and seeded on 96-well plates at a density of 1 × 10^4^ in 200 µL complete medium/well. After 24 h, during which HeLa and CaSki cells adhered to the surface of the plates, treatments with functionalized gold nanoparticles and biocompounds and/or doxorubicin were applied, with a dilution of 1/20. Increasing concentrations of doxorubicin were used to compare the effects of nanoparticles with standard treatments in the human clinic as follows: 2.1 µg/mL (a similar concentration to the therapeutic one used in human clinics) and two higher concentrations of 6.25 µg/mL and 12.5 µg/mL. The experiments were performed in triplicate. The viability of cells was evaluated after 24 and 48 h by an MTT assay that uses a tetrazolium salt (3-(4,5-dimethylthiazolyl-2)-2,5-diphenyltetrazolium bromide). MTT salt is enzymatically reduced by viable cells into formazan. After 24 and 48 h of exposure to treatments, the medium was extracted from the wells, 100 µL of MTT solution (1 mg/mL)/well was added, and the plates were incubated for 1 h at 37 °C in the dark. The MTT solution was discarded from the wells, and 150 µL of DMSO/well was added to solubilize the formazan crystals from the cells. Optical density readings were performed at 492 nm using a BioTek Synergy 2 microplate reader (Winooski, VT, USA). The cytotoxic effect of doxorubicin and/or of biomolecules, such as resveratrol, was expressed as the relative viability (% control) and calculated as follows. The percentage of cell survival in the negative control (i.e., untreated cells) was assumed to be 100%. Relative viability = [(experimental absorbance − background absorbance)/(absorbance of untreated controls − background absorbance)] × 100%.

MTT assay for DF MSCs is given in “Effects of functionalized GNPs on healthy dental follicle mesenchymal stem cells (DF MSCs)”, Supplementary Material.

#### 2.5.8. Statistical Analysis

The data are presented, from triplicate experiments, as mean ± standard deviation. Statistical analysis was performed with the GraphPad Prism 5 program, using two statistical analysis methods: *one-way ANOVA* followed by the “Dunnett’s Multiple Comparison Test” posttest for the comparison of the treated cells with the control sample without treatment, setting the *p*-value at *p* < 0.05 (statistically significant), *p* < 0.01, or *p* < 0.001 (highly statistically significant). The *t*-test for the comparison of two samples with significant values set for *p* < 0.05 (*). Significances were thus noted in the graphic representations: ^ns^ *p* > 0.05, * *p* < 0.05, ** *p* < 0.01, or *** *p* < 0.001. The results of the *one-way ANOVA* statistical analysis followed by the Dunnett posttest are represented in graphs by red stars; the *t*-test results are positioned above the blue brackets that indicate the pairs of samples analyzed.

## 3. Results and Discussion

### 3.1. Characterization of the GNP_R and GNP_R1 in Colloidal Systems

During the synthesis of the GNP_R, the color of the reaction mass instantly changed to red, indicating the formation of GNPs with their characteristic color due to surface plasmon resonance (SPR). The centrifuged and washed GNP_R was then redispersed in bi-distilled water (pH 5.6) resulting in GNP_R1, in colloidal dispersion, having the same Au content as in synthesized GNP_R. The SPR band in the visible domain of the UV-VIS spectrum, with the maximum intensity of the absorption peak at 535–538 nm, is shown in Figure 1. It is important to note that the UV-VIS spectrum is nearly the same for both GNP_R and GNP_R1 colloidal dispersions, which contain the same Au content. As a result, in the graphical presentation, only GNP_R1 is shown, which is further functionalized with several biomolecules, D, R, P, and Ic, as revealed in the legend of Figure 1.

**Figure 2:**
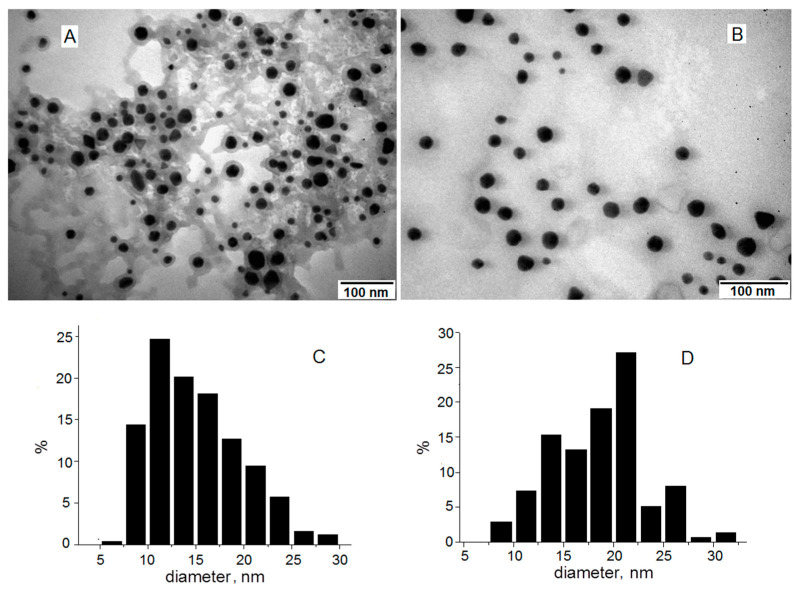
TEM images for GNP_R (**A**) and GNP_R1 (**B**). The bars in the images are 100 nm. Histograms of size distribution for GNP_R, the average size, d, is 14.0 ± 4.7 nm (**C**), and GNP_R1, d, is 17.5 ± 4.9 nm (**D**).

TEM images of initial GNP_R in aqueous colloidal solution, as a result of synthesis (panel A), and GNP_R1, the centrifuged and washed GNPs, redispersed in bi-distilled water, and diluted to the same concentration, of 0.91 mM (179 mg/L Au) in colloidal solution, as in the initial one (panel B) are given in Figure 2.

The GNP_R and GNP_R1 particles in the TEM picture had approximately spherical shapes. Centrifugation and washing eliminated the majority of the organic material generated during the synthesis, as seen in the TEM image (Figure 2A), around GNP_R and among nanoparticles, leaving only the capping layer on the GNP_R1 particles, as shown in Figure 2B. GNP_R and GNP_R1 are gold nanoparticles that have been coated with resveratrol during their manufacture and may be referred to as unfunctionalized. Later, the GNP_R1 particles (unfunctionalized or as manufactured) are functionalized with anticancer medicines such as doxorubicin, as well as different biomolecules with documented anticancer activity such as piperine (P), resveratrol (R), and/or icariin (Ic). The diameters of a large number of particles (several hundred) were measured on different TEM images for GNP_R and GNP_R1, and the histograms of size distribution are represented in Figure 2C and Figure 2D, respectively.

The average diameter of GNP_R1 particles is 17.5 ± 4.9 nm (Figure 2D), somewhat higher than GNP_R (14.0 ± 4.7 nm, Figure 2C), likely due to the loss of the smallest GNPs in the supernatant after centrifugation. On the contrary, the average hydrodynamic diameter determined by dynamic light scattering (DLS) for GNP_R1 (46.8 nm) is smaller than that for GNP_R (63.3 nm) (the corresponding DLS plots are given in Figure S1A,C, Supplementary Material). The size of the nanoparticles measured by DLS is larger than the diameters estimated by TEM, since TEM pictures measure the actual diameter of the GNP (core), whereas DLS obtains a hydrodynamic diameter that encompasses the GNP (core) and its organic capping layer. Thus, washing the centrifuged GNPs and subsequent GNP_R1 results in a significant reduction in DLS diameter due to the partial removal of the organic coatings, which include oxidation products from synthesis and are distributed across GNP_R (Figure 2A). Washing reduces the zeta potential of GNP_R1 particles to −16.8 mV, compared to −20.7 mV for GNP_R, as seen from the corresponding zeta-potential plots in Figure S1B,D in Supplementary Material. It is consequently insufficient to ensure electrostatic protection against aggregation. The threshold value for electrostatic stability is ±30 mV. As a result, the organic coating layer on the gold nanoparticles plays a significant role in providing protection.

The high stability of this GNP_R1 dispersion was confirmed by UV-VIS spectra, which remained unmodified for months, as shown in Figure S2 for UV-VIS spectra plotted at times from 30 min to 6 months, since preparation (Supplementary Material). This is due largely to the protection by resveratrol coating layer adsorbed on Au surface of particles. The R coating layer is formed from self-assembled resveratrol molecules, through hydrogen bonds and electrostatic interactions [7].

**Figure 3:**
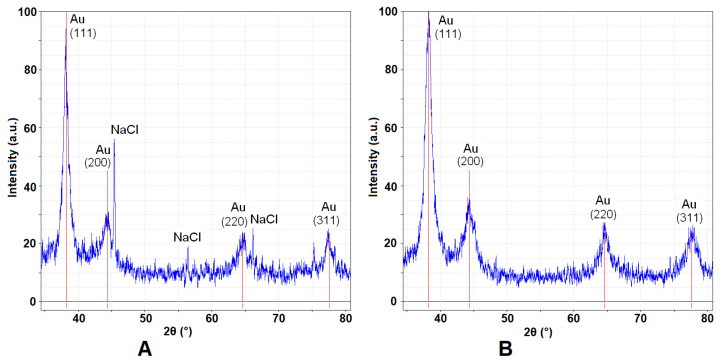
X-ray diffraction patterns for GNP_R (**A**) and GNP_R1 nanoparticles (**B**); [00-0000] experimental blue curves; PDF: [89-3697] of the red vertical bars give the positions of diffraction lines for ordered crystalline Au domains.

The X-ray diffraction patterns for gold nanoparticles are shown in Figure 3. The GNP_R peaks for gold crystallite domains are evidenced (Figure 3A) along with peaks for NaCl (resulting from the synthesis reaction between HAuCl_4_ and resveratrol in alkaline medium, NaOH). However, in the XRD diffraction pattern (Figure 3B) for GNP_R1, the peaks for NaCl are no longer present, due to washing after centrifugation of gold nanoparticles.

Gold crystallites have distinct peaks at 2θ(°) values: 38.22(°) corresponds to the (111) plane, the strongest signal; 44.42(°) to the (200) plane; 64.64(°) to the (220) plane; and 77.85(°) to the (311) plane. So, the presence of crystalline gold is confirmed. The NaCl peaks occur as a result of the reaction between HAuCl_4_ and resveratrol in an alkaline media (NaOH). The NaCl peaks are clearly proven at 2θ(°) values: 45.66(°) corresponds to the (220) plane, 56.74(°) to the (222) plane, and 66.56(°) to the (400) plane.

By using the Scherrer’s equation at a 2θ(°) value, of about 38.22 (°) for the (111) plane, the average size of ordered crystallite domains in synthesized GNP_R nanoparticles was found to be about 9.4 nm (Figure 3A) and 8.3 nm for crystallite domains size in GNP_R1 nanoparticles (Figure 3B). The average size of GNP_R nanoparticles, calculated using Scherrer’s equation on the Au (111) diffraction peak displayed in Figure 3A, is around 9.4 nm, which is nearly comparable to the mean size of 8.3 nm for GNP_R1, centrifugated and washed Au nanoparticles (Figure 3B). These values are significantly smaller than the observed value from TEM images, as seen in Figure 2. This is because the size of ordered crystalline domains is calculated and is obtained within the gold nanoparticles by XRD. Usually, these values are smaller than the size of nanoparticles.

**Figure 4:**
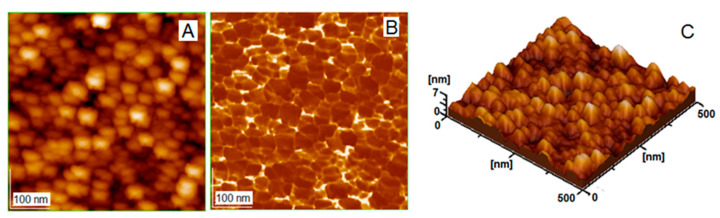
AFM images of GNP_R1: 2D topography (**A**); phase image (**B**); 3D topography (**C**); scanned area 500 nm × 500 nm. Surface roughness as root mean square, RMS, is given as 3.5 nm.

The surface morphology of GNPs spread on solid support is shown in Figure 4, by AFM.

Figure 4 displays 2D topography (A), a phase image (B), and 3D topography (C) for almost spherical nanoparticles of GNP_R1, purified by centrifugation and washing, and stabilized by a resveratrol shell. The core of gold nanoparticles is covered with a layer of resveratrol, R, evidenced by a yellow color in the phase image (B). The shape of the particles resembles a spherical form, as determined through TEM images. The particle diameter is estimated as the full width at half maximum of the profile peak height, to eliminate the convolution effect of the AFM probe’s tip shape with the surface characteristics of the shell layer, as shown by us previously [71]. By measuring a statistical number of approximately 30 particle sizes, an average diameter (d) can be estimated for particles of GNP_R1.

The diameter of nanoparticles for GNP_R1 is 24.9 ± 4.1 nm, as shown in the AFM image, Figure 4A (e.g., about four nanoparticles can be counted on the bar length of 100 nm, as given in image A). Furthermore, the root mean square (RMS) value is used to compute the surface roughness value [68,69,70]; see the 3D image in panel C of Figure 4. The roughness value of 3.5 ± 1.3 nm was generated by AFM software (AFM Win SPM2.0 Processing software, JEOL, Tokyo, Japan) and shows that the adsorbed layer of GNP_R1 nanoparticles on the glass plate is very well spread and has a low RMS value. Thus, AFM topographies also revealed that these gold nanoparticles are very well spread within the adsorbed layer on the glass surface, showing directly that they were also very well dispersed in highly stabilized colloidal solution.

### 3.2. GNP_R1 Interaction with Drugs

**Figure 5:**
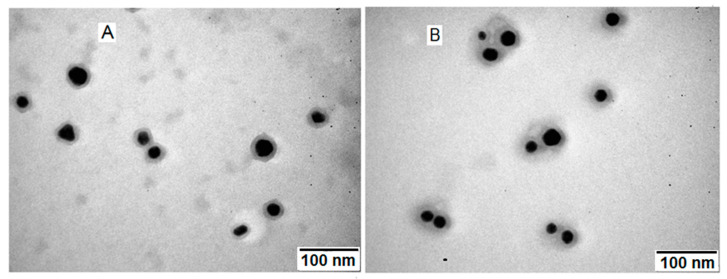
TEM images for functionalized GNP-R1, used in composition (**9**), Table 2: GNP_R1 4.8 µg/mL, Ic 1.3 µg/mL, and D 2 µg/mL, GNP_R1@Ic/D; composition (**10**), Table 3: GNP_R1 3.6 µg/mL, R 0.14 µg/mL, P 0.35 µg/mL, Ic 0.7 µg/mL, and D 2 µg/mL, GNP_R1@R/P/Ic/D; the bar is 100 nm (**A**,**B**). The averaged size, d, of multi-functionalized GNPs is about 19.4 ± 5.1 nm.

The GNP_R1 pellet, centrifuged and washed and then diluted in PBS, shows a behavior similar to that of GNP_R in its interaction with the anticancer compounds in aqueous solutions of doxorubicin, resveratrol, piperine, and icariin. The strongest effect on the UV-VIS absorption band in the 2:1 *v*/*v* mixtures of GNP_R1 and the different compounds (given in Figure 1) is manifested for icariin (Ic) solution and doxorubicin (D) solution, where an evident flattening of the SPR peak is evident. This effect of doxorubicin is reduced by adding R, P, and/or Ic solution; these compounds stabilize the mixture containing doxorubicin, as shown in TEM images (Figure 5).

**Figure 6:**
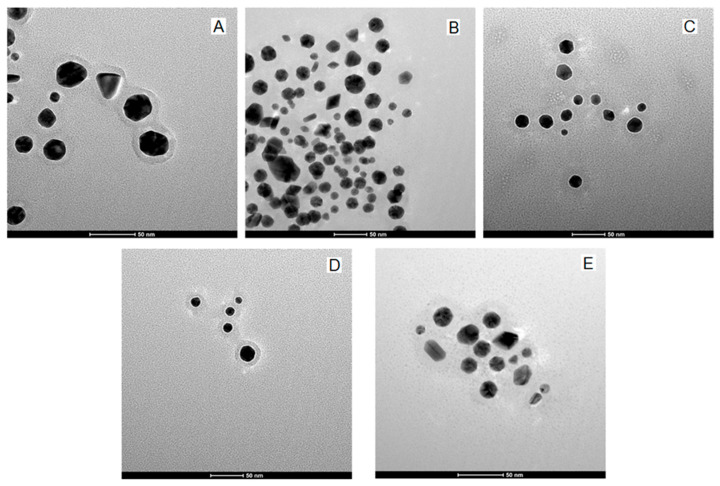
HR-TEM images of (**A**) GNP_R1 with doxorubicin (D) (4/1 *v*/*v*), containing 50 mg/L Au, 2.9 mg/L of D; (**B**) GNP_R1 (50 mg/L) and resveratrol 6 mg/L; (**C**) GNP_R1 (50 mg/L) with piperine (4 mg/L); (**D**) GNP_R1 (50 mg/L) with icariin (6.2 mg/L); (**E**) a mixture containing GNP_R1 (50 mg/L), resveratrol (6 mg/L), piperine (4 mg/L), icariin (6.2 mg/L), and doxorubicin (1 mg/L). The bars are 50 nm.

From Figure 5, it is clear that GNP-R1/D nanoparticles can be further stabilized by using biomolecules as capping agents, such as in composition 9, GNP-R1/D/Ic (also symbolized as GNP_R1@D/Ic), Figure 5A, and composition 10, GNP_R1/R/P/D/Ic (e.g., GNP_R1@R/P/D/Ic), Figure 5B. These coatings prevent aggregation by keeping the nanoparticles separate from one another and in consequence, enhancing their stability. Definitely, through engineering strategy, the properties of GNPs can also be improved by molecular functionalities of these capping biomolecules, realizing that the shell morphology of these drug carriers have important roles in their interaction with biological membranes and targeting cancer cells. For medical-specific applications, the GNP_R1 surface must be functionalized with various biomolecules—like D, R, P, and Ic, as in composition 10, GNP-R1/R/P/D/Ic, Figure 5B, that selectively might participate in specific processes—in our experiments to carry the doxorubicin and increase its anticancer activity inside the cancer cells, in the absence of side effects on healthy cells. The general mechanism of GNP stabilization involves charge stability on the nanoparticle surface achieved by adsorption of charged biomolecules as a function of medium pH, which can help nanoparticles repel each other from becoming aggregated or prevent them from touching each other by steric effects. The chosen biomolecules, R, P, and Ic, and their mixture revealed that they can be used as an adsorbed layer on the GNP-R1@D surface for protection from agglomeration (see Figure 5 and Figure 6). The functionalized gold nanoparticles might develop self-assemblies of various nanostructure morphologies on the cancer cell membrane, helping processes that kill cancer cells. In addition, they are biocompatible (Table S1 and MTT cell viability assay, Figure S3, Supplementary Material) and biodegradable and do not promote cytotoxicity in healthy cells at the concentrations used in this work. Consequently, the biocompatibility of these nanocomposites was tested on adult dental follicle mesenchymal stem cells (DF MSCs) [67]. The MTT cell viability assay was performed to monitor the response of DF MSCs to GNP_R1 and GNPs functionalized with resveratrol, piperine, icariin, and/or doxorubicin. The results showed a complete lack of cytotoxicity. Thereby, GNP_R1 and GNP_R1 functionalized with biomolecules, GNP_R1@R/P/Ic, or with doxorubicin, GNP_R1@D, did not influence the growth and proliferation rate of the healthy DF MSCs. The characteristics of these human stem cells and the MTT cell viability experiments are shown in Figure S3 and Table S1, Supplementary Material.

For the HR-TEM images, the solutions of GNP_R1 and the solutions of anticancer compounds were mixed and diluted with PBS to obtain the concentrations given below.

HR-TEM images for the GNP_R1@D mixture containing 50 mg/L (253.8 µmol/L) Au and 2.9 mg/L (5.34 µmol/L) doxorubicin clearly show the shapes of particles and their organic coating layer (Figure 6A). For this low content of doxorubicin (molar ratio Au/D = 47/1), no visible aggregation of the particles appears.

HR-TEM images of GNP_R1 mixtures, with resveratrol and with the other biomolecules and doxorubicin, unveil the effects of therapeutic drugs (Figure 6A–E), which are similar, revealing high stability in the colloidal solutions of these functionalized GNPs. Some associations of these gold nanoparticles mediated by the added biocompounds are visible, particularly in the presence of icariin (Figure 6D,E). However, the functionalized gold nanoparticles remain well dispersed and evidently separated. In the sample containing GNP_R1 and icariin alone, the HR-TEM image (Figure 6D) shows the initial stages of self-assembly formation of these nanoparticles. Despite this, the functionalized gold nanoparticles remain very well separated, with a stabilized capping layer individually very well evidenced and clearly visible on their surfaces. This characteristic makes them as promising nanostructures for use in bioengineered devices, potentially offering unique optical and electrical properties for medical applications.

#### 3.2.1. Stability of GNPs in Aqueous Dispersion

The stability of GNPs in dispersion is crucial in their use as carriers in biomedicine. Factors, like surface modification of GNPs, pH, temperature, and ionic strength, all influence the stability of GNPs, preventing them from aggregating or degrading. Stabilization strategy of GNPs, using capping agents like D, R, P, and/or Ic, is essential to maintain the desired size of nanoparticles and optimal stable dispersion of multi-functional GNPs in aqueous solutions. To better control the pH conditions during the functionalization process of GNP_R1 with doxorubicin, the formation of GNP_R1@D was accomplished in pure (bi-distilled) water of initial pH of 5.6. The pH was measured with a pH meter and only a small variation in pH (5.8 ± 0.2) was observed. The formation of GNP_R1@D was also accomplished in PBS (pH 7.4), and the stability of functionalized gold nanoparticles was not affected by pH value. Furthermore, the formation of a unique self-assembled (coating) layer of biomolecules (R and D) on GNPs, and its high stability, was confirmed by SPR absorption band (in Figure S2, Supplementary Material) as well as DLS and zeta potential measurements. The interaction mechanism of D and R, within the self-assembled layer adsorbed on AuNPs, can be explained through hydrogen bonds and electrostatic interactions. Multi-functionalization of GNPs with R, P, Ic, and D resulting in GNP_R1@D/R/P/Ic was also realized in PBS (pH 7.2 ± 0.2) and in aqueous solutions (pH 5.8–7.2).

The enhanced stability of these dispersions is achieved through various mechanisms, including the high stability of self-assembled layer of capping molecules on GNPs, as well the electrostatic repulsion and steric hindrance between nanoparticles. The improved stability of multi-functional GNPs is realized primarily through their surface functionalization by using the said capping agents as a self-assembled layer adsorbed on AuNPs.

#### 3.2.2. Enhanced Stability of Multi-Functional GNPs in Colloidal Solutions

In the following experiments, the nanoparticles of original GNP_R1 and functionalized GNPs were diluted in volume ratios of 1:1, 1:2, and also 2:1 with PBS (pH 7.4) or DMEM (pH 7.2–7.4). Different samples were kept at room temperature (around 20 °C) for 15 min; then, the size of gold nanoparticles in colloidal solutions was measured by DLS, and UV-VIS absorption spectra were recorded. One example, GNP_R1/PBS 2/1 *v*/*v* ratio (119.3 mg/L, Au), is shown in Figure 1. The SPR band of these nanoparticles does not show any change in peak shift or broadening by dilution of original nanoparticles GNP_R1 in PBS. Overall, original GNP_R1 and multi-functional GNPs with selected diverse coatings, in biological buffers, like PBS or DMEM medium, displayed similar results, indicating very well-stabilized nanocarriers for working in cervical cancer, HeLa and CaSki, cell lines.

The hydrodynamic sizes of nanoparticles dispersed in PBS, starting with the original GNP_R1, about 47 nm, to the multi-functionalized GNPs, like GNP_R1@R/P/D/Ic, around 50 nm, were determined by DLS measurements. An example is given in Figure S1, panel E, for GNP_R1@R, with an average DLS size of 49.6 nm, Supplementary Material. The synthesized resveratrol-coated GNPs, noted GNP_R1, and functionalized nanoparticles with other coatings were very stable, displaying less than 10% oscillations in sizes. Clearly, the multi-functional GNPs are highly stabilized in the colloidal solutions. They also demonstrated resistance to high ionic strength aqueous environments, like PBS and DMEM. Through TEM, AFM, and HR-TEM measurements, the same size values for GNP_R1 (core) 17.5 nm and for functionalized GNPs (core/shell) of about 40 nm were obtained, demonstrating the stabilized size of these nanocarriers in aqueous dispersions of bi-distilled water (pH 5.6) to PBS (pH 7.4).

In the mixture of original GNP_R1 colloidal solution with solution of resveratrol in PBS (Table 1), for a 2:1 volume ratio (Figure 1), the GNP_R1@R nanoparticles were successfully formed. This was demonstrated by the SPR band; the zeta potential value for GNP_R1@R is −33.4 mV, as viewed in Figure S1F (Supplementary Material), compared to −16.8 mV, for original GNP_R1, is due to the functionalization of gold nanoparticles and the contribution of negative charge on resveratrol molecules. In the mixture of GNP_R1 colloidal solution with solution of resveratrol in PBS and solution of doxorubicin in water (Table 1), at a 2:1:1 volume ratio (Figure 1), the GNP_R1@R/D nanoparticles were prepared in colloidal solution. The zeta potential value for GNP_R1@R/D nanoparticles is about −28 mV, compared to −16.8 mV, for original GNP_R1, due to the contribution of negative charge from resveratrol molecules and positive charge of doxorubicin molecules. In the mixture of GNP_R1 colloidal solution with solution of resveratrol in PBS and solution of piperine in PBS and solution of doxorubicin in water, at a 2:1:1:1 volume ratio (Figure 1), the GNP_R1@R/P/D was synthesized in colloidal solution. The zeta potential value for GNP_R1@R/P/D is −29.8 mV, compared to −16.8 mV for GNP_R1, due to the contribution of negative charge from resveratrol molecules and positive charge of doxorubicin molecules, and piperine is an uncharged molecule.

Furthermore, the zeta potential, from −20 mV for original GNP_R1 in PBS, to −28 mV for GNP_R1@R/D, to −30 mV for GNP_R1@R/P/D/Ic, shows an increased negative value for multi-functional GNPs. Clearly, a progressive enhanced stability of colloidal dispersions is evidenced by functionalization of original GNP_R1 nanoparticles, reaching the electrostatic stability threshold of ±30 mV, at pH 7.4. Moreover, GNP_R1@R particles disclosed a zeta potential of −33.4 mV in PBS. Definitely, the highly stable self-assembled layers coating GNPs are formed primarily through hydrogen bonds and electrostatic interactions.

### 3.3. Potential Anticancer Efficacy of Functionalized GNPs on Human Cancer Cells

**Figure 7:**
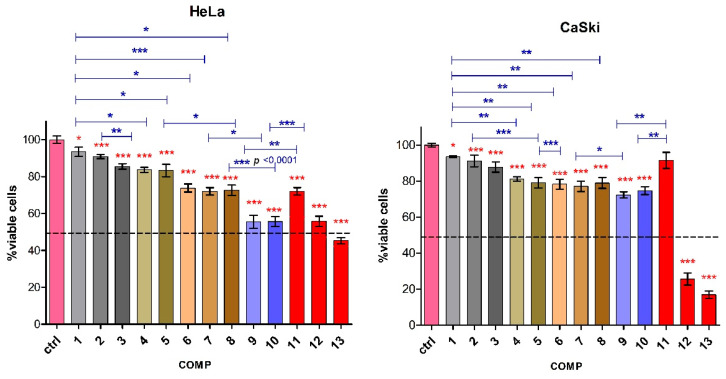
Control (ctrl): untreated cells; 1–13 sample compositions are disclosed in Table 2. Statistical analysis representation: *one-way ANOVA* results are illustrated with red stars and *t*-test results with blue brackets and blue stars, for HeLa and CaSki cells, at 24 h. The *p*-values are set * *p* < 0.05, ** *p* < 0.01, or *** *p* < 0.001.

Beneficial effects of functionalized GNPs were examined using a comparative investigation on HeLa and CaSki cell viability in response to GNP_R1 alone and functionalized GNPs with resveratrol, piperine, and icariin +/− doxorubicin. In this study, the effects on cervical carcinoma cells’ viability were investigated as a response to cells’ treatment with ten compositions, including GNP_R1 alone and functionalized GNP_R1 as a comparison term with three concentrations of doxorubicin alone. The concentration of doxorubicin in the compositions varied between 0.42 µg/mL and 2 µg/mL, as shown in Table 2. The differences between GNP_R1 alone and GNP_R1 functionalized with biocompounds (R, P, or Ic) +/− doxorubicin were statistically evaluated (Figure 7).

The results demonstrated that GNP_R1 (alone, sample 1) and GNP_R1 functionalized with resveratrol 0.5 µg/mL (sample 2) are not harmful, with a viability of around 93% for HeLa cells and 90% for CaSki cell lines. Adding 0.7 µg/mL doxorubicin (sample 3) reduced cell proliferation by 14% for HeLa cells and 13% for CaSki. The GNP_R1 functionalization with resveratrol (0.38 µg/mL) and doxorubicin (0.52 µg/mL), as in sample 4, produced a substantial rise in cytotoxicity, more pronounced for the CaSki line, up to 20%. The conclusion was that incorporating resveratrol and doxorubicin generated a stronger toxicity than GNP_R1 alone. The GNP_R1 is also simplified noted as GNP.

Concerning the functionalization of GNP with piperine, GNP-P, a more pronounced cytotoxic effect of 22% was recorded in CaSki cells. For instance, in GNP-P 0.66 µg/mL (sample 5) and GNP-P 0.53 µg/mL-D 0.42 µg/mL (sample 8), this beneficial cytotoxic effect was intensified, especially for the CaSki line at the value of 28%, but it only slightly influenced the HeLa line.

GNPs functionalized with icariin, GNP-Ic 1.66 µg/mL (sample 6), had the strongest cytotoxic effect on the HeLa line (27%) and less on the CaSki line (22%), and the introduction of doxorubicin at a concentration of 0.42 µg/mL, as in GNP-Ic 1.3 µg/mL-D 0.42 µg/mL (sample 7), enhanced cells’ response. But when doxorubicin at 2 µg/mL concentration was added into the GNP-P, GNP-P 0.53 µg/mL-D 2 µg/mL (sample 10), and GNP-Ic complexes, GNP-Ic 1.3 µg/mL-D 2 µg/mL (sample 9), this cytotoxic effect profoundly increased to 45% for HeLa cells and to 28% for the CaSki line, even at a smaller quantity of Au. It is noteworthy that the HeLa line is more sensitive to the doxorubicin alone of 2.1 µg/mL with a cytotoxicity value of 29%, while the CaSki line was relatively resistant to this concentration of doxorubicin (cytotoxicity index of 9%) (Figure 7). At a higher dose of doxorubicin (samples 12 and 13), the CaSki line was more sensitive.

Sample 9—with mole ratios of Au/D 6.63, Au/Ic 12.7, and D/Ic 1.92, very close to those of sample 10 (Au/D 6.63, Au/P 13.1, and D/P 1.98)—unveiled significantly higher cytotoxicity as compared to sample 11, concerning doxorubicin alone at a D concentration much higher than in the other two systems. The drug delivery systems containing multi-functionalized gold nanoparticles (samples 9 and 10) might have a potential application due to a synergistic effect of all components leading to a high level of combined cytotoxic activity, particularly on the HeLa cell line. The high cytotoxicity of samples 9 and 10 might be attributed to the size of the particles, GNP-R1/P/D (GNP_R1@P/D) and GNP-R1/Ic/D (GNP_R1@Ic/D), and to biomolecules P or Ic associated with D at particular mole ratios, which enhanced the interaction with biological membranes. This effect might also be described by the synergism between doxorubicin and GNP_R1@P or between doxorubicin and GNP_R1@Ic, resulting in an enhanced facility exerted by the nanoparticles in the intracellular transport of doxorubicin.

Figure 7 shows the comparison between HeLa and CaSki cell lines’ response concerning cell viability to the different treatments: GNPs alone, GNPs functionalized with resveratrol, piperine, and icariin, with or without doxorubicin, and doxorubicin alone.

The statistical analysis of data from Figure 7, using one-way ANOVA, showed highly significant differences compared to the untreated control (ctrl sample) for all compounds tested for both cell lines, except for unfunctionalized GNP, sample 1, and for the lowest dose of doxorubicin alone for the CaSki cell line. Also, the *t*-test used for comparison between selected compounds, especially between GNP alone and the other compositions, revealed the induction of a statistically significantly increased cytotoxicity of different levels.

GNP alone (sample 1) did not show cytotoxicity on cervical cancer cells, but the addition of a very small dose of doxorubicin (sample 3) induced a decrease in cell proliferation. Similar results related to the lack of toxicity of GNPs are reported in the literature. Venditti et al. (2020) showed that neither AuNP concentration (between 1.0 and 45.5 µg/mL) nor GNPs were able to trigger a stress-dependent signal transduction pathway with further activation of apoptotic cascade [72]. In the same study, the authors compared the anticancer effects of hydrophilic gold nanoparticles (AuNPs), obtained by adding resveratrol during AuNPs synthesis (AuNPs@RSV1), and resveratrol alone. Resveratrol decreased the breast cancer MCF-7 cell number at only 10 µM concentration, while AuNPs@RSV1 decreased cell number already at 1.0 µM in a dependent manner, suggesting that the association with AuNPs could enhance cell entrance of resveratrol [72].

The antiproliferative effects of resveratrol–gold nanoparticles compared with resveratrol nanoemulsion were reported in a pancreatic cancer model using BxPC-3 cells and pancreatic islet endothelial cell line MS1 [73]. A dose-dependent decrease in viability was observed for the MS1 and BxPC-3 cells after receiving treatment with the R-GNPs at a 2.5–20 µg/mL dosage (resveratrol) and 6.66–52.78 µg/mL (gold), and comparing resveratrol alone and R-GNP at the same doses revealed that incorporation of resveratrol induced a higher toxicity than GNPs. The proposed cellular mechanisms of action of R-GNPs and resveratrol are related to the cell apoptosis processes with down-regulation of cyclin A, cyclin B, CDK1, and CDK2 expression and up-regulation of p53 and p21 expressions and decreasing *Bcl-2* expression [73].

The apoptotic response of hepatocarcinoma cells HepG2 was reported at 8 µg/mL concentrations of R-GNP, more pronounced than that of resveratrol alone at the same concentration. These data confirmed that Res-GNPs induced a higher antitumor effect than resveratrol alone in vitro and in vivo, due to gold nanoparticles’ ability to carry additional resveratrol into cells and locate in mitochondria [74].

The cellular internalization of resveratrol–GNPs was evaluated on MDA-MB-231 breast, PANC-1 pancreatic, and PC-3 prostate cancer cell lines. Dark-field microscopic images showed that resveratrol–GNPs were internalized into tumor cells after only 2 h of administration, with the best cellular uptake taking place at 4–24 h of administration. The increased resveratrol corona of resveratrol–GNPs showed a superior cellular uptake after 24 h [75].

Use of R-GNPs as drug carriers was performed on human glioma cells (LN229 cells) by Mohanty et al. (2014), revealing the dose-dependent cytotoxicity of R-GNPs, doxorubicin-loaded R-GNPs, and doxorubicin alone, determined after 48 h [38]. An enhanced antitumor effect was observed for doxorubicin-loaded R-GNPs, with an IC50 value of 4 µg/mL compared with an IC50 of doxorubicin alone at 6 µg/mL [38], which can be explained by the synergism between doxorubicin and R-GNPs and the facility exerted by the nanoparticles in the intracellular transport of doxorubicin.

Loading anticancer drugs onto a nanocarrier can aid in the discharge of drug molecules, the endocytosis process being a very dynamic and energy-dependent process [76]. Endocytosis and drug release also depend on nanoparticle characteristics (surface charge, size, shape, and hydrophobicity), cell type, and expression of cell receptor type and density [76]. Thus, the effect of doxorubicin and cisplatin was greatly potentiated by the conjugation of these drugs to GNPs in a study on human osteosarcoma cells MG-63 and Saos-2 by delivering drugs into the nucleus of cancer cells through caveolae-dependent endocytosis. A dose-dependent response was observed, with the highest doxorubicin–GNP concentration of 1 µg/mL inducing a 90% cytotoxic response [76].

In our study, among the biocompounds used as conjugates, resveratrol and piperine added to the GNP composition had similar effects of inducing cytotoxicity, which was significantly increased by the presence of doxorubicin. Icariin induced more intense effects compared to resveratrol and piperine, especially on the HeLa cell line. Increasing the dose of doxorubicin to 2 µg/mL from GNP complexes functionalized with piperine and icariin induced the highest values of cell death on the HeLa cell line. Piperine belongs to a family of nitrogenous aggregates obtained from black pepper (*Piper nigrum*), with a historical healing approach conferred by its antioxidant, anti-inflammatory, neuroprotective, anticancer, anti-microbial, hepatoprotective, and cardioprotective effects. Its medical usage is limited because of its low solubility in water and reduced bioavailability. Piperine loading in nanoparticles could be beneficial as a targeted drug delivery system because piperine can bypass the outflow of molecules by P-glycoprotein 1 (P-gp) and thus control the multidrug resistance in cancerous cells [77]. Piperine- and curcumin-loaded PEG-PLGA nanoparticles were used for MCF-7 breast cancer treatment with enhanced cytotoxic response due to piperine’s ability to act as both a bioenhancer of curcumin activity and as an anticancer drug [78]. Likewise, in another study on MCF-7 cells, piperine-loaded polymeric (piperine-encapsulated polycaprolactone) nanoparticles with high concentration (100 µg/mL) were used in comparison with free piperine, and a significant difference was observed in the cytotoxic response. This higher cytotoxicity of piperine nanoparticles was made possible by the nanocarrier’s prolonged release and increased cellular uptake in tumor cells [79]. Piperine-loaded GNPs were developed in several studies but without addressing the anticancer effects. Black pepper AuNPs showed significant in vivo anti-inflammatory, analgesic, and sedative potential at much lower doses than the free biocompound [8,80]. Another application of piperine-coated GNPs was in the prevention of insulin aggregation [81] or combating neurotoxicity induced by paraquat [82].

Icariin and its derivative icaritin are known for their pharmacological properties, such as anti-osteoporosis, anti-inflammation, anti-oxidative stress, anti-depression, and antitumor activity. The biological effects of icariin at doses of 20 µM in cervical cancer are exerted by activating the *mTOR/PI3K/AKT* signaling pathways with the induction of apoptosis and autophagy [83,84]. Icariin alone at a 20 µg/mL dose can reverse the drug chemoresistance of tumor cells, as demonstrated by Jiang et al. (2019) [85] in a study on the cisplatin-resistant SKVCR ovarian cell line. Icariin repressed cell viability and autophagy but stimulated G0/G1 phase cell cycle arrest and apoptosis [85]. In a similar manner, icariin repressed the growth of MCF-7 cells and also has a probable function that defeats tamoxifen resistance in MCF-7/TAM cells using doses between 10 and 75 µM [86]. In nano-formulation, icariin was used in the therapy of osteoporosis and osteogenic repair as icariin-loaded selenium–gold multi-shell nanocomposites or electrospun icariin-loaded core/shell collagen, polycaprolactone, and hydroxyapatite composite scaffolds [87,88].

In light of these data, in the present experiments, differences were observed between two lines of cervical carcinoma, with a more efficient response of HeLa cells to the GNP systems functionalized with biocompounds. The functionalization (conjugation) of GNP_R1 with icariin/piperine and doxorubicin at very low doses induced a cytotoxic response similar to the 2 µg/mL dose of doxorubicin, suggesting better internalization of the drug in cancer cells. The association of GNP_R1 nanoparticles conjugated with icariin (sample 9) or piperine (sample 10) with the dose of 2 µg/mL of doxorubicin increased the cytotoxic response of HeLa cells at the dose of 6.25 µg/mL of doxorubicin alone (sample 12).

### 3.4. Cellular Viability in Response to Doxorubicin Alone or Combined with Natural Molecules and to Functional Gold Nanoparticles at 24 and 48 h

Our study investigated the extent to which doxorubicin alone or combined with biocompounds (natural molecules)—as well as gold nanoparticles (GNPs) functionalized with these compounds and each loaded with an equivalent dose of doxorubicin (2 µg/mL)—affect the viability of HeLa and CaSki tumor cells, following 24 and 48 h of exposure. The various combinations of biocompounds, GNP-R1, and doxorubicin are detailed in Table 3.

**Figure 8:**
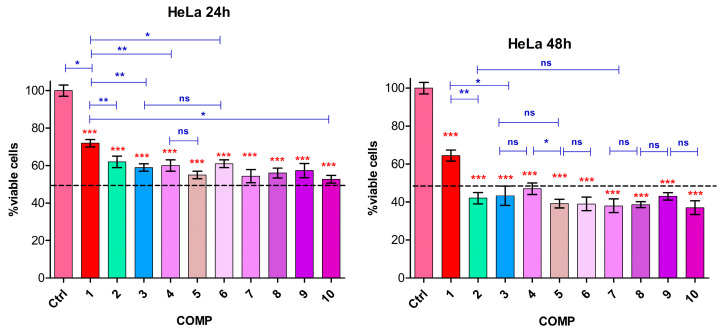
Control (Ctrl): untreated cells; 1–10 sample compositions are disclosed in Table 3. Statistical analysis representation: *one-way ANOVA* results are illustrated with red stars and *t*-test results with blue brackets and blue stars, at 24 h and 48 h, on HeLa cells. The *p*-values are set ^ns^ *p* > 0.05, * *p* < 0.05, ** *p* < 0.01, or *** *p* < 0.001.

**Figure 9:**
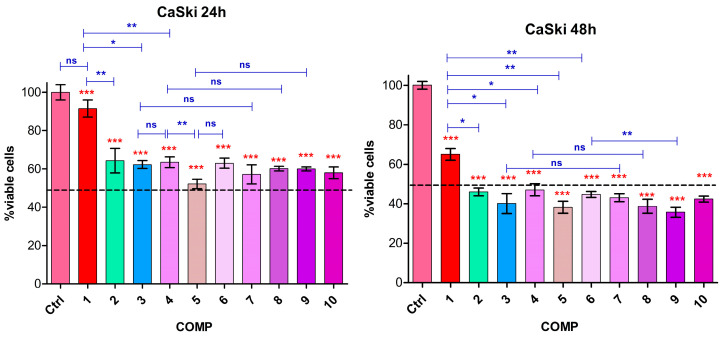
Control (Ctrl): untreated cells; 1–10 sample compositions are disclosed in Table 3. Statistical analysis representation: *one-way ANOVA* results are illustrated with red stars and *t*-test results with blue brackets and blue stars, at 24 h and 48 h, on CaSki cells. The *p*-values are set ^ns^ *p* > 0.05, * *p* < 0.05, ** *p* < 0.01, or *** *p* < 0.001.

The results demonstrate that HeLa cells are more sensitive to doxorubicin (2 µg/mL, sample 1), exhibiting 29% cytotoxicity after 24 h and 38% after 48 h (Figure 8). In contrast, CaSki cells showed only a 10% reduction in viability at 24 h, increasing to 34% at 48 h (Figure 9). Comparable cytotoxic effects (~38–40% for HeLa, ~36–38% for CaSki) were observed after 24 h for combinations of doxorubicin with resveratrol (sample 2), piperine (sample 3), and their mixture (sample 4). The combination of Ic (1.0 µg/mL) with doxorubicin (sample 5) resulted in the highest cytotoxicity after 24 h ~45% for HeLa and ~48% for CaSki. These effects intensified at 48 h, with cytotoxicity reaching ~55% for samples 2 and 3, and up to 60% for sample 5 and samples 6–10, which include various GNP-R1 formulations with biocompounds and doxorubicin, in both cell lines. Notably, GNPs functionalized with doxorubicin alone (sample 6) enhanced cytotoxicity by ~10% in HeLa and ~28% in CaSki cells compared to doxorubicin alone at 24 h. At 48 h, these differences decreased. Functionalization of GNP-R1 with biocompounds and doxorubicin (samples 7–9) further amplified cytotoxicity, with the highest effect in HeLa observed for sample 10 (GNP-R1 3.6 µg/mL, R 0.14 µg/mL, P 0.35 µg/mL, Ic 0.7 µg/mL, D 2 µg/mL), and in CaSki for sample 9 (GNP-R1 4.8 µg/mL, Ic 1.0 µg/mL, D 2 µg/mL), both reaching approximately 65% cytotoxicity (Figure 8 and Figure 9).

Compositions 9 and 10 are a result of comparing different compositions and various measurements to assess which is the best from the observed data for a particular cancer cell line. For instance, the increased cytotoxic response to comp. 10 for HeLa cell line can be explained by the increased stability of advanced gold nanoparticles by selecting appropriate concentrations of gold, doxorubicin, and biomolecules (GNP_ R1 3.6 μg/mL, R 0.14 μg/mL, P 0.35 μg/mL, Ic 0.7 μg/mL, D 2 μg/mL). The choice of concentration of D, R, P, and Ic in the composition 10 was assessed and selected by different measurements, e.g., UV-VIS absorption spectra as shown in Figure 1. This specific coating with biomolecules and doxorubicin on GNP_R1 core nanoparticles could prevent their aggregation and enhance their stability and anticancer efficacy.

Figure 8 shows the percentage of viable HeLa cells (relative to the control, based on the MTT assay) as a function of composition at 24 h and 48 h.

Figure 9 displays cell viability (viable cells in % of control, from MTT assay) versus composition for CaSki cells at 24 and 48 h.

Different sensitivity of the two cell lines to doxorubicin, at the dose of 2 µg/mL, was observed. The synergistic response in terms of cytotoxicity was observed for biocompounds combined with doxorubicin, with more intense effects on the HeLa line at 24 h and a progressive increase at 48 h (Figure 8). A similar effect was found for the CaSki line, but with a lower intensity, especially at 48 h (Figure 9). The addition of biocompounds and doxorubicin in the nanoparticle structure caused a moderate decrease in viability for both tumor lines compared to the cytotoxic response to doxorubicin alone, much more visible at 48 h, suggesting a better bioavailability of the drug induced by the presence of biocompounds. The highest cytotoxicity levels were observed with the GNP_R1 and icariin, as well as the associations of all biocompounds and doxorubicin.

In Figure 8 and Figure 9, the statistical analysis with *one-way ANOVA* revealed an important decrease in cell viability compared to untreated HeLa and CaSki control cells, with highly significant differences; the *t*-test showed a significant difference between doxorubicin alone versus biocompounds combined with the same dose of doxorubicin.

In both cervical carcinoma cell lines, the cytotoxic response was significantly enhanced when doxorubicin (2 µg/mL) was combined with biocompounds compared to treatment with doxorubicin alone at the same therapeutic concentration. No statistically significant differences were observed among the various biocompounds tested. Notably, this potentiation effect became more pronounced at 48 h post-treatment. In this study, we chose to monitor the response to cells’ treatment at 48 h, because the duration of late apoptotic response can take from 3–4 h to 24–48 h, depending on cell type and specific features like caspase-3 activation, and *bcl-2* expression. The synergistic enhancement of doxorubicin’s cytotoxic activity by such biocompounds has been well documented in the scientific literature. The low cytotoxicity of resveratrol was reported by Al-Abd, A.M., et al. 2011 [89] in a study on three tumor cell lines (MCF-7, HepG2, and HeLa cells). Association of resveratrol with doxorubicin and docetaxel induced increased cytotoxicity for both antitumor drugs. The synergy between resveratrol and drugs was induced by inhibition of p-glycoprotein efflux and decreasing of *MDR1* gene expression [89]. Another advantage of resveratrol in combination with doxorubicin is its cardioprotective effect, which can counteract the cardiotoxicity induced by doxorubicin [90,91]. In another study, Jurkat leukemic lymphocytes medicated with barasertib/everolimus together with resveratrol showed an increased synergistic cytotoxic response as an effect of inducing apoptosis, enlarged quantities of hydroperoxide, and minor modifications in protein carbonyl products, with the mention that none of these parameters were found in normal lymphocytes [92]. Piperine appears to have similar mechanisms to resveratrol in the synergistic action with cytotoxic drugs, as demonstrated by a study on MDCK-MDR1 kidney cells, transfected with the *MDR1* gene, treated with piperine and docetaxel. Microarray examination has shown that piperine subdued P-gp and *CYP1B1* gene expression and considerably changed the expression of genes implicated in inflammatory response, angiogenesis, cell proliferation, or cell migration [93]. Also, piperine has a direct anticancer effect at higher concentrations (100 µM) by inhibiting TGF-β signaling pathways and interfering with epithelial–mesenchymal transition (EMT) events in HepG2, MDA-MB-231, and A549 cell lines [94]. Icariin has direct apoptotic effects on wide cancer cells, with an increase in the *Bax*/*Bcl2* ratio, release of cytochrome c, cleavage of poly(ADP-ribose) polymerase, and activation of caspases [95]. The icariin effect of sensitization of the resistant osteosarcoma doxorubicin (DOX)-resistant cell line MG-63/DOX at a non-toxic concentration of doxorubicin was reported by Wang Z et al. (2015) [56]. Icariin increased intracellular doxorubicin retention and accumulation with blockage of the activity of MDR1 protein and enhanced doxorubicin-induced apoptosis [56]. Higher doses of icariin (40–160 μg/mL) suppressed cell growth and induced apoptosis in human gallbladder carcinoma cell lines GBC-SD and SGC-996 in a dose-dependent manner. A dose of 40 µg/mL icariin enhanced the antitumor properties of gemcitabine (0.5 μmol/L) in cell lines by inhibiting gemcitabine-induced NF-κB activity and improving caspase-3 activity, inducing G(0)-G(1) phase arrest, and inhibiting the expression of *Bcl-2*, *Bcl-xL*, and surviving proteins [96].

**Figure 10:**
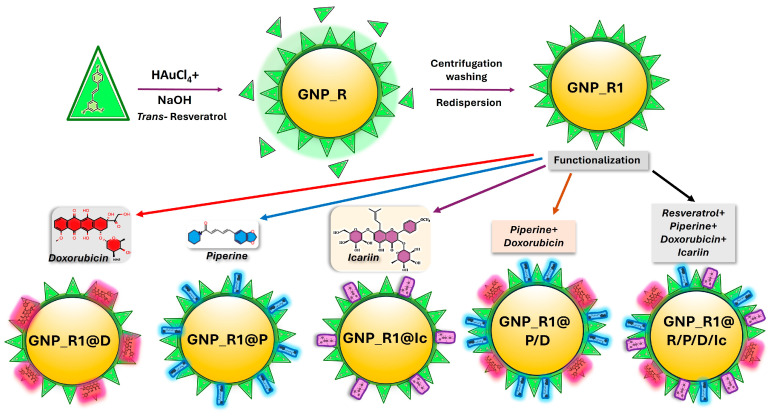
Schematic representation of the surface modification evolution of gold nanoparticles, GNPs, synthesized in this work by green synthesis employing tetrachloroauric acid as a precursor and trans-resveratrol, R, as a reducing agent. GNPs coated with a resveratrol capping layer, denoted GNP_R, were purified further by centrifugation and washing, and the resulting pellet of GNPs was redispersed in bi-distilled water and in various biological media, PBS or DMEM, to produce centrifuged and washed gold nanoparticles, denoted GNP_R1. The surface modification of gold nanoparticles is generated through non-covalent interactions of gold nanoparticles with biomolecules, R, P, and/or Ic, and doxorubicin, D, resulting in a self-assembled layer on the nano-gold surface.

Gold nanoparticles (GNP_R1) were functionalized with resveratrol (R, 0.5 µg/mL) obtaining GNP_R1@R, with doxorubicin (D, 0.7 µg/mL) resulting in GNP_R1@D, piperine (P, 0.66 µg/mL) obtaining GNP_R1@P, and icariin (Ic, 1.66 µg/mL) resulting in GNP_R1@Ic, corresponding to samples 2, 3, 5, and 6 in Table 2. A more complex system (sample 10, Table 3) was developed via a self-assembled layer adsorbed on Au surface. Using an advanced approach, by incorporating resveratrol, piperine, doxorubicin, and icariin, with final concentrations of GNP_R1 (3.6 µg/mL), R (0.14 µg/mL), P (0.35 µg/mL), D (2 µg/mL), and Ic (0.7 µg/mL), the GNP_R1@R/P/D/Ic was obtained, as illustrated in Figure 10. This multicomponent nanoparticle formulation exhibited the highest stability and an enhanced cytotoxicity against cervical cancer, HeLa and CaSki, cells, likely due to the synergistic effects of the combined bioactive agents with doxorubicin and gold nanoparticles.

The biomolecules, R, P, Ic, and doxorubicin, can interact by intermolecular hydrogen bonds and electrostatic interactions within the capping self-assembled layer on the gold surface, leading to enhanced functionalized GNP stability. So, the self-assembled layer is provided by non-covalent interactions of gold nanoparticles with the biomolecules and doxorubicin [97,98]. The high stability of these advanced nanocarriers in colloidal solution is maintained by repulsive forces, to overcome van der Waals interactions and assure the electrostatic repulsion, among the functional gold nanoparticles. The GNPs are highly stable due to the presence of high surface charges, which prevent the agglomeration of functionalized GNPs.

## 4. Conclusions

This research study demonstrates that aqueous solutions, at a constant concentration of D (2 μg/mL) plus R (0.5 μg/mL) and P (0.5 μg/mL) and/or Ic (1.0 μg/mL) gradually decreased the viability of HeLa and CaSki cells at 24 h and even more after 48 h. So, these data show the anticancer effect of natural compounds, R, P, and Ic, supporting the enhanced anticancer activity of D.

Further aqueous colloidal solutions of functional GNP_R1 (e.g., GNP_R1@R/P/Ic/D) at the same constant concentration in doxorubicin (2 μg/mL), sharply decreased the viability of HeLa and CaSki cells at 24 h, even more for 48 h, at lower concentrations in R (0.2 μg/mL), P (0.4 μg/mL), and/or icariin (0.7 μg/mL). Moreover, this result highlights the importance of functional GNPs, mainly GNP_R1@R/P/Ic, as a general platform for doxorubicin delivery. In particular, due to their high zeta potential values for GNP_R1@R/P/Ic and GNP_R1@R, they simultaneously ensure the enhanced stability of these novel GNP_R1@R/P/D/Ic carriers. Due to their demonstrated anticancer efficacy on human cervical cancerous, HeLa and CaSki, cells, they might be beneficial in advanced treatment of cervical cancer.

### Future Perspectives

Our in vitro study demonstrated the biocompatibility of gold carrier nanoparticles in combination with natural chemicals R, P, and Ic, GNP_R1@R/P/Ic, on cervical cancer, HeLa and CaSki, cell lines utilizing the MTT assay. The enhanced cytotoxic activity of doxorubicin loaded on GNP_R1@R/P/Ic against human cervical cancer cells showed the suitability of these GNP_R1@R/P/Ic nanocarriers for anticancer drugs. In vitro assessment demonstrated that the advanced GNP_R1@D/R/P/Ic nanoparticles exhibited remarkable stability and significant anticancer efficacy against cervical cancer cells, with few anticipated harmful side effects.

Future in vivo animal research will ascertain the robust safety profile of these nanoparticles, with enhanced blood circulation, less drug leakage, and extended circulation time of doxorubicin within the body. In vivo experiments are also crucial to determine the GNP_R1@D/R/P/Ic nanoparticles’ biological half-life and evaluate their biotoxicity.

## Figures and Tables

**Table 1:** Solutions used in this study.

Solution	Solvent	Chemical Formula	Molar Mass (g/mol)	Concentration	
				mg/L	mmol/L
GNP_R, GNP_R1	Water	Au	197	179	0.91
Doxorubicin, D	Water	C_27_H_29_NO_11_	543.5	42	0.077
Resveratrol, R	PBS	C_14_H_12_O_3_	228.2	30	0.131
Piperine, P	PBS	C_17_H_19_NO_3_	285.3	40	0.140
Icariin, Ic	DMSO, PBS	C_33_H_40_O_15_	676.7	100	0.148

**Table 2:** Shows the type of sample/composition, revealed as concentrations in µg/mL and in µmol/L, i.e., µmolar concentrations of gold (Au), doxorubicin (D), resveratrol (R), piperine (P), and icariin (Ic), and the mole ratios of Au/D, Au/R, Au/P, Au/Ic, D/R, D/P, D/Ic, R/P, R/Ic, and P/Ic for each sample (1–13).

Samples (COMP)	Concentration µmol/L					Mole Ratios
Concentration µg/mL	Au	D	R	P	Ic	
1—GNP_R1 9.1	46.2	-	-	-	-	-
2—GNP_R1 5.9, R 0.5	29.9	-	2.19	-	-	Au/R 13.7
3—GNP_R1 5.9, D 0.7	29.9	1.29	-	-	-	Au/D 23.2
4—GNP_R1 4.5, R 0.38, D 0.52	22.8	0.96	1.66	-	-	Au/D 23.8; Au/R 13.7; D/R 0.58
5—GNP_R1 5.9, P 0.66	29.9	-	-	2.31	-	Au/P 12.9
6—GNP_R1 5.9, Ic 1.66	29.9	-	-	-	2.45	Au/Ic 12.2
7—GNP_R1 4.8, Ic 1.3, D 0.42	24.4	0.773	-	-	1.92	Au/D 31.6; Au/Ic 12.7; D/Ic 0.40
8—GNP_R1 4.8, P 0.53, D 0.42	24.4	0.773	-	1.86	-	Au/D 31.6; Au/P 13.1; D/P 0.416
9—GNP_R1 4.8, Ic 1.3, D 2	24.4	3.68	-	-	1.92	Au/D 6.63; Au/Ic 12.7; D/Ic 1.92
10—GNP_R1 4.8, P 0.53, D 2	24.4	3.68	-	1.86	-	Au/D 6.63; Au/P 13.1; D/P 1.98
11—D 2.1	-	3.86	-	-	-	-
12—D 6.25	-	11.5	-	-	-	-
13—D 12.5	-	23.0	-	-		-

**Table 3:** Shows the type of sample/composition, the concentration in µg/mL and in µmol/L, i.e., µmolar concentration of gold (Au), doxorubicin (D), resveratrol (R), piperine (P), and icariin (Ic), and the mole ratios of Au/D, Au/R, Au/P, Au/Ic, D/R, D/P, D/Ic, R/P, R/Ic, and P/Ic for each sample (1–10).

Samples (COMP)	Concentration, µmol/L					Mole Ratios
Concentration, µg/mL	Au	D	R	P	Ic	
1—D 2	-	3.68	-	-	-	-
2—R 0.2, D2	-	3.68	0.88	-	-	D/R 4.18
3—P 0.5, D 2	-	3.68	-	1.75	-	D/P 2.10
4—R 0.5, P 0.5, D 2	-	3.68	2.19	1.75	-	D/R 1.67; D/P 2.10; R/P 1.25
5—Ic 1, D 2	-	3.68	-	-	1.48	D/Ic 2.49
6—GNP_R1 4.8, D 2	24.4	3.68	-	-	-	Au/D 6.63
7—GNP_R1 4.8, R 0.2, D 2	24.4	3.68	0.88	-	-	Au/D 6.63; Au/R 27.7; D/R 4.18
8—GNP_R1 4.8, P 0.5, D 2	24.4	3.68	-	1.75	-	Au/D 6.63; Au/P 13.9; D/P 2.10
9—GNP_R1 4.8, Ic 1, D 2	24.4	3.68	-	-	1.48	Au/D 6.63; Au/Ic 16.5; D/Ic 2.49
10—GNP_ R1 3.6, R 0.14, P 0.35, Ic 0.7, D 2	18.3	3.68	0.61	1.23	1.03	Au/D 4.97; Au/R 30.0; Au/P 14.9; Au/Ic 17.8; D/R 6.03; D/P 2.99; D/Ic 3.57; R/P 0.50; R/Ic 0.59; P/Ic 1.19

## Data Availability

Data are contained within the article and Supplementary Materials.

## Supplementary Materials

The following supporting information can be downloaded at: https://www.mdpi.com/article/10.3390/biomedicines13081861/s1, Figure S1. DLS plots for (A) GNP_R: Z-average size, PDI; (C) GNP_R1: Z-average size, PDI; (E) GNP_R1@R: Z-average size, PDI and zeta-potential plots for (B) GNP_R; (D) GNP_R1; (F) GNP_R1@R. Figure S2. UV-VIS spectra of GNP_R1 colloidal solution after 1 h since preparation, after 7 days, 3 months and 6 months. Figure S3. MTT cell viability assay results for dental follicle MSCs (DF MSCs) treated with GNP_R1 and GNPs functionalized with biomolecules, R, P, and/or Ic and doxorubicin, D. Table S1 shows the type of sample/composition, revealed as the concentrations in µg/mL and in µmol/L, i.e., µmolar concentrations of gold (Au), doxorubicin (D), resveratrol (R), piperine (P), and icariin (Ic) and the mole ratios of Au/D, Au/R, Au/P, Au/Ic, D/R, D/P, D/Ic, R/P, R/Ic and P/Ic for each sample (1–7).

## Abbreviations

The following abbreviations are used in this manuscript:

D	doxorubicin
R	trans-resveratrol
P	piperine
Ic	Icariin
PBS	phosphate-buffered saline
GNPs	gold nanoparticles
GNP_R	gold nanoparticles coated with a resveratrol layer
GNP_R1	centrifuged and washed gold nanoparticles
GNP_R1@D/R	gold nanoparticles two-functionalized
GNP_R1@D/R/P	gold nanoparticles three-functionalized
GNP_R1@D/R/P/Ic	gold nanoparticles multi-functionalized
SPR	surface plasmon resonance
TEM	transmission electron microscopy
XRD	X-ray diffraction
AFM	atomic force microscopy
UV-VIS	ultraviolet–visible spectroscopy
HR-TEM	high-resolution transmission electron microscopy
HPV	human papillomavirus
DLS	dynamic light scattering
ζ-potential	zeta potential
MTT	cell viability assay
