# Supplementary material for: Enhanced Stability of Multi-Functionalized Gold Nanoparticles and Potential Anticancer Efficacy on Human Cervical Cancer Cells
Source: Biomedicines. 2025 Jul 31;13(8):1861. doi: 10.3390/biomedicines13081861 (PMC12383472; doi:10.3390/biomedicines13081861)
Supplement: Supplementary file 1 [file biomedicines-13-01861-s001.zip › biomedicines-3737752-supplementary.pdf]

Supplementary material

Type of manuscript: *Article*
Title: Enhanced Stability of Multi-Functionalized Gold Nanoparticles and Potential Anticancer Efficacy on Human Cervical Cancer Cells

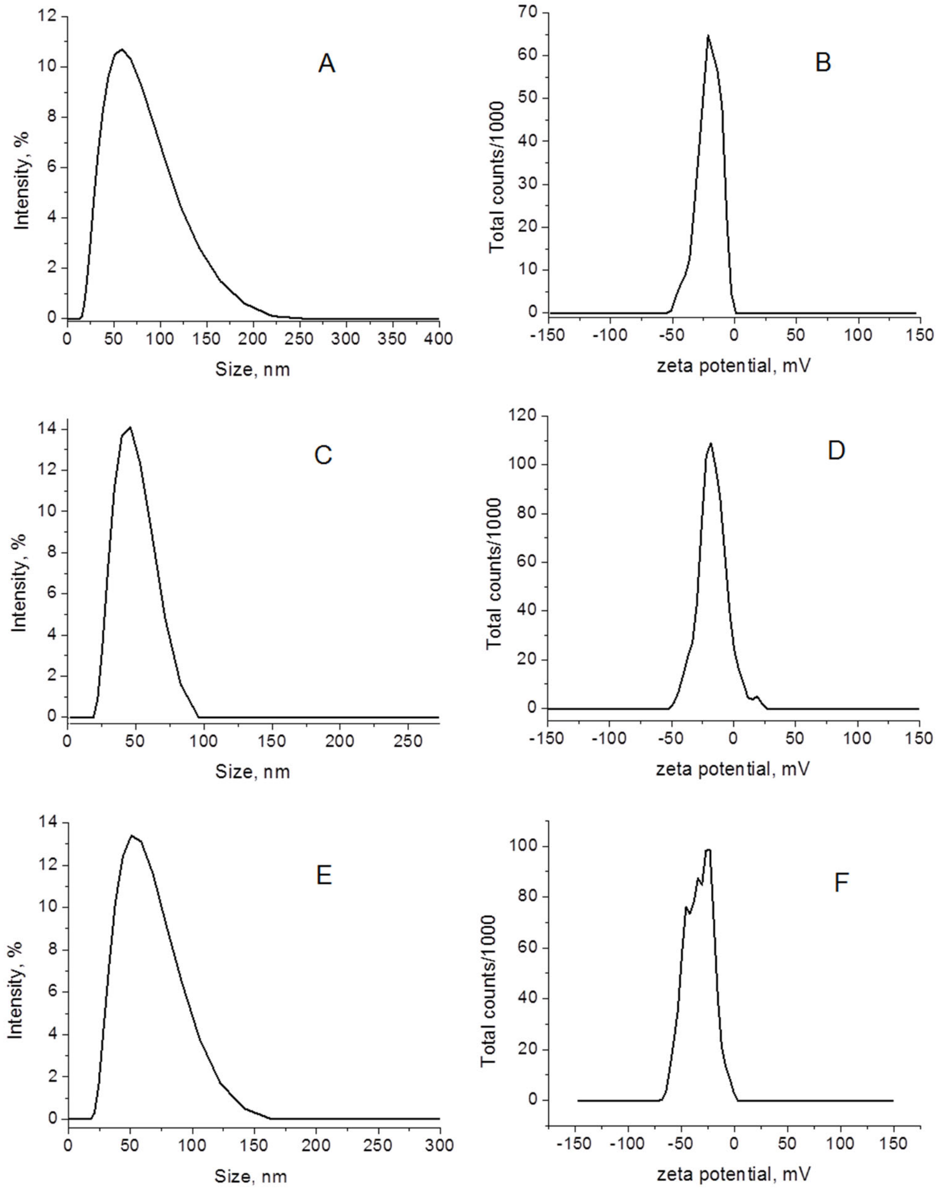

Figure S1. DLS plots for (**A**) GNP_R: Z-average size 63.29 nm, PDI 0.477; (**C**) GNP_R1: Z-average size 46.77 nm, PDI 0.311; (**E**) GNP_R1@R: Z-average size 49.58 nm, PDI 0.428 and zeta-potential ζ plots for (**B**) GNP_R: ζ = -20.7 mV; (**D**) GNP_R1: ζ = −16.8 mV; (**F**) GNP_R1@R: ζ = −33.4 mV (PDI is the polydispersity index of the particles size distribution).

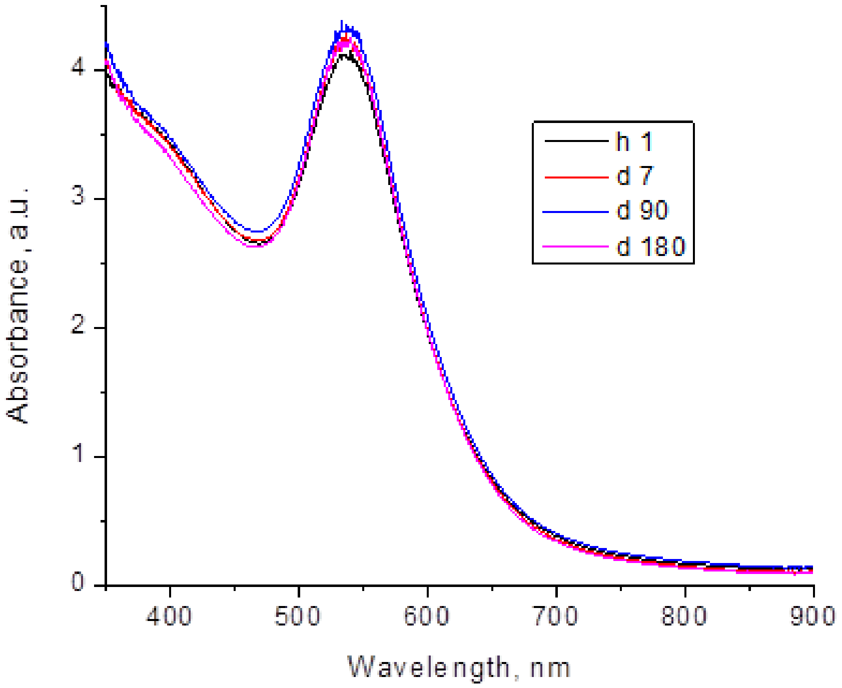

Figure S2. UV-VIS spectra of GNP_R1 colloidal solution after 1 hour since preparation, after 7 days, 3 months and 6 months.

Effects of functionalized GNPs on healthy dental follicle mesenchymal stem cells (DF MSCs)

To evaluate the biological effects of natural compounds (resveratrol, R, piperine, P and icariin, Ic), doxorubicin, D, synthesized gold nanoparticles, GNP_R1, and composite nanoparticles, GNP_R1@R/P/Ic/D, on healthy (DF MSCs) cells, adult human mesenchymal stem cells isolated from the dental follicle (DF) were used. The clinical protocol of isolation and the patients' informed consent was approved by The Ethical Committee of the Iuliu Hatieganu University of Medicine and Pharmacy, Cluj-Napoca, registration number 292/6.05.2011. These cells (named **Dental Follicle MSCs** or **DF MSCs**) express STRO-1, Oct3/4, Sox-2, Nanog, Notch1; they were negative for markers of hematopoietic lineage CD34 and CD117, and positive for mesenchymal stem cell markers CD44, CD29, CD 90 and CD105. Also, the ability to differentiate into different cell lineages (osteoblasts, adipocytes and neuron-like cells) has been demonstrated [67] (the reference is also given in the article). Cultivation medium consisted by Dulbecco's modified Eagle's medium (DMEM) high glucose/F-12HAM (Sigma) containing 15% fetal calf serum (FCS, Sigma), 2 mM L-Glutamine, 1% antibiotics, 1% non-essential aminoacids (NEA), 55 μM beta-mercaptoethanol, 1 mM natrium piruvate (all reagents from Sigma-Aldrich

reagents). The DF MSCs were cultivated in 96-well plates, with a seeding cell density of 10^4 cells/well in 200 µL complete medium/well. The treatments with the selected compounds (Table S1) were administered after 24 hours at a final dilution of 1/20. Each treatment and control were carried in biological triplicates. MTT assay was performed after 24 hours of exposure. The results are illustrated in Figure S3. One-way ANOVA followed by the "Dunnett's Multiple Comparison Test" post-test for the comparison of the treated cells with the control sample without treatment, setting the *p*-value at $p < 0.05$ (statistically significant), $p < 0.01$, or $p < 0.001$ (highly statistically significant). The t-test for the comparison of two samples with significant values were set for $p < 0.05$ (GraphPad 5.0 Software, San Diego, CA, USA). As shown in Figure S3, DF-MSCs growth and proliferation of nanoparticle treatments were very similar to untreated control cells. No statistical differences were observed between untreated control cells and those treated cells with GNP_R1 and functional gold nanoparticles, suggesting the biocompatibility of GNP_R1 and GNP_R1 functionalized with R, D, P or Ic. The only statistical difference observed was between comp. **1** (GNP_R1 9.1 µg/mL) and comp. **6** (GNP_R1 4.8 µg/mL, Ic 1.3 µg/mL, D 0.42 µg/mL, noted as GNP_R1@Ic/D, where the *t*-test revealed a $p < 0.01$, signifying that GNPs functionalized with icariin and doxorubicin, GNP_R1@Ic/D, might have a slight inhibitory effect on DF MSCs growth.

Table S1. shows the type of sample/ composition, revealed as the concentrations in µg/mL and in µmol/L, i.e., µmolar concentrations of gold (Au), doxorubicin (D), resveratrol (R), piperine (P), and icariin (Ic) and the mole ratios of Au/D, Au/R, Au/P, Au/Ic, D/R, D/P, D/Ic, R/P, R/Ic and P/Ic for each sample (1–7); (numbers from brackets denotes the corresponding composition from Table 2, in the article, used to treat DF MSCs, healthy cells).

**Samples (COMP)**	**Concentration, µmol/L**					**Mole Ratios**
**Concentration µg/mL**	**Au**	**D**	**R**	**P**	**Ic**	
1 - GNP_R1 9.1; (**1**)	46.2	-	-	-	-	-
2 - GNP_R1 5.9, D 0.7; (**3**)	29.9	1.29	-	-	-	Au/D 23.2
3 - GNP_R1 4.5, R 0.38, D 0.52; (**4**)	22.8	0.96	1.66	-	-	Au/D 23.8; Au/R 13.7; D/R 0.58
4 - GNP_R1 5.9, P 0.66; (**5**)	29.9	-	-	2.31	-	Au/P 12.9
5- GNP_R1 5.9, Ic 1.66; (**6**)	29.9	-	-	-	2.45	Au/Ic 12.2
6 - GNP_R1 4.8, Ic 1.3, D 0.42; (**7**)	24.4	0.773	-	-	1.92	Au/D 31.6; Au/Ic 12.7; D/Ic 0.40
7 - GNP_R1 4.8, P 0.53, D 2; (**10**)	24.4	3.68	-	1.86	-	Au/D 6.63; Au/P 13.1; D/P 1.98

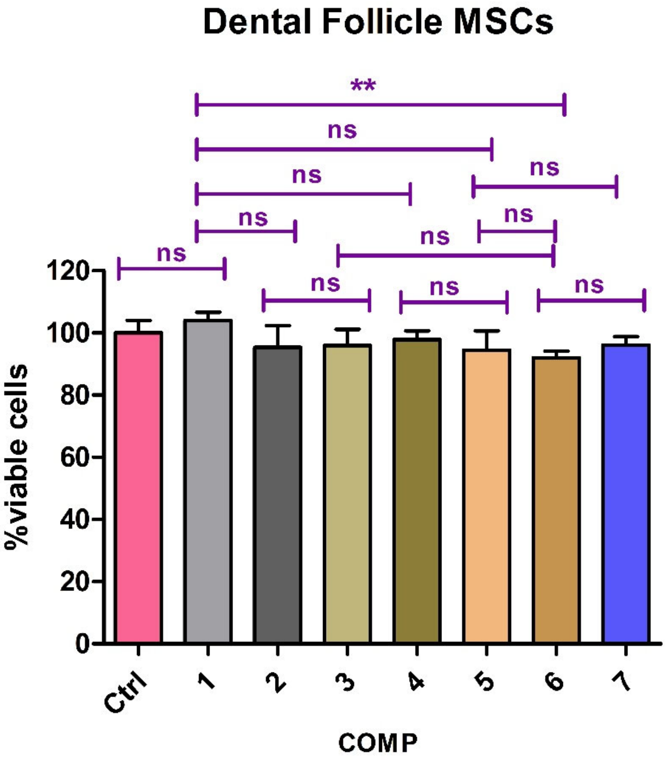

Figure S3. MTT cell viability assay results for dental follicle MSCs (DF MSCs) treated with GNP_R1 and GNPs functionalized with biomolecules, R, P, and/or Ic and doxorubicin, D.

Legend: Ctrl — untreated cells; 1 — GNP_R1 9.1 µg/mL; 2 — GNP_R1 5.9 µg/mL, D 0.7 µg/mL; 3 — GNP_R1 4.5 µg/mL, R 0.38 µg/mL, D 0.52 µg/mL; 4 — GNP_R1 5.9 µg/mL, P 0.66 µg/mL; 5 — GNP_R1 5.9 µg/mL, Ic 1.66 µg/mL; 6 — GNP_R1 4.8 µg/mL, Ic 1.3 µg/mL, D 0.42 µg/mL; 7 — GNP_R1 4.8 µg/mL, P 0.53 µg/mL, D 2 µg/mL Statistical analysis representation: *t*-test results with purple brackets and stars. The *p*-values are set ^{ns} $p > 0.05$, * $p < 0.05$, ** $p < 0.01$, or *** $p < 0.001$.

Reference

[67] Lucaciu O.; Soriţău O.; Gheban D.; Ciuca D.R.; Virtic O.; Vulpoi A.; Dirzu N.; Câmpian R.; Băciuţ G.; Popa C.; Simon S.; Berce P.; Băciuţ M.; Crisan B. Dental follicle stem cells in bone regeneration on titanium implants. *BMC Biotechnol*. **2015,** *15,* 114. doi: 10.1186/s12896-015-0229-6
